# Nursing Must Lead From Its Own Knowledge: A Tribute to Jacqueline Fawcett

**DOI:** 10.1155/jonm/2645505

**Published:** 2026-07-07

**Authors:** Leah Korkis, K. David Bailey, Maria W. O’Rourke

**Affiliations:** ^1^ Keck Medicine of USC, Los Angeles, California, USA; ^2^ UCLA Health, Los Angeles, California, USA, uclahealth.org; ^3^ Maria W. O′Rourke & Associates, LLC, Larkspur, California, USA

Jacqueline Fawcett, PhD, RN, SnD (hon), FAAN, ANEF, one of nursing’s most consequential scholars, passed away in March 2026. Fawcett retired in 2025 after more than 45 years of distinguished service to the broader UPenn Philadelphia and UMass Boston communities located in the United States. Over her career, she published more than 300 journal articles and book chapters and authored foundational texts translated into multiple languages. This editorial is a tribute to her memory and to the clarity, courage, and stewardship she brought to nursing’s intellectual life.

## 1. Her Ensuring Gift to Nursing

Fawcett gave “Nursologists,” a term she coined to represent scholars and practitioners, more than influential concepts. She gave the nursing discipline a clearer way to understand itself. Across decades of scholarship, teaching, mentorship, and public intellectual stewardship, she returned repeatedly to one essential claim: nursing is not sustained by goodwill, labor, or institutional presence alone. It is sustained by a distinct body of knowledge, and that knowledge must be named, used, developed, and defended. This claim remains relevant to nurse leaders because it shapes how nursing is perceived within institutions and underscores why professional role identity formation matters. In many organizations, leaders work within systems shaped by financial imperatives, biomedical priorities, regulatory requirements, and managerial frameworks developed outside nursing. Such realities are unavoidable. Her work did more than organize nursing knowledge; it disciplined nursing’s self‐understanding. She asked the profession to be precise when vagueness was easier, to be courageous when borrowed language was safer, and to be accountable to its own intellectual tradition.

Fawcett’s stewardship of nursing knowledge also extended to her foundational role in https://nursology.net, which she co‐founded in 2018 as a living repository of nursing discipline–specific knowledge. Developed and maintained by volunteer nurse scholars, the site provides access to resources on nursing conceptual models, theories, philosophies, methodologies, organizations, conferences, publications, and ongoing work in practice, research, education, and health policy. Through her substantial contributions to its theory content, resources, and blog dialog, Fawcett helped make nursing knowledge visible, accurate, and available for ongoing disciplinary dialog.

Fawcett was a formidable scholar and steward of the discipline, and her death is a great loss. It does not lessen the urgency of her message; it sharpens it. The question she leaves with nursing leadership is not whether nursing participates in complex health systems; it clearly does. The question is whether nursing will participate from a position of intellectual clarity that differentiates and strengthens its contribution.

## 2. From Metaparadigm to Leadership

Fawcett’s articulation of nursing’s metaparadigm gave the discipline a lasting way to name its central concerns: person, health, environment, and nursing, later expanded to include culture. Across the years, her scholarship consistently emphasized the metaparadigm’s role in defining the boundaries and focus of the discipline. In other words, nursing is not only defined by the tasks nurses perform but also by the phenomena that demand attention and the purposes that give the discipline its form and meaning. This is not simply a curricular point; it is a leader’s responsibility.

For nurse leaders, the responsibility is higher and extends beyond preserving professional language or theoretical alignment. It requires intentional action to embed nursing’s disciplinary perspective into operational decisions, care delivery models, quality initiatives, and organizational culture. Leadership choices that fail to reflect nursing’s unique lens risk reducing nursing practice to a series of transactional tasks rather than a professional discipline grounded in theory, concepts, and application, including contextual understanding.

Professional practice models, governance structures, and strategic plans must reflect a distinct nursing perspective, grounded in the discipline’s literature and professional role obligations. A nurse leader’s responsibility becomes particularly important with professional governance structures where nurse leaders shape how nursing knowledge is translated into standards and daily practice. Fawcett’s work emphasized that the metaparadigm defines the focus and boundaries of nursing knowledge, helping preserve the discipline’s distinct professional role identity.

Grounded by Fawcett’s view that the metaparadigm defines the boundaries and scope of nursing knowledge and must guide practice, administration and professional development. As a result, this lens underscores the uniqueness and diversity of each individual. Nurse leaders work in differently structured health services, under different regulatory regimes, and with different workforce pressures. Yet across those differences, the same principle applies: nursing leaders must be able to explain how nurses understand the people they serve, the environments in which care occurs, the health processes nursing seeks to influence, the work of nursing itself, and the cultural contexts that shape all of these dimensions.

## 3. The Conceptual–Theoretical–Empirical (CTE) Structure and the Visibility of Nursing’s Contributions

One of Fawcett’s most practical contributions for contemporary nursing leadership is the CTE structure. The power of the CTE structure lies in its insistence that nursing activities should be traceable to a nursing conceptual model, specified through theory, and made visible through congruent empirical indicators. For nurse leaders, the practical value of this structure is perhaps most evident in quality improvement initiatives. Typically, improvement work is conducted as an operational exercise: solve a local problem, improve a metric, and move on. Fawcett offered a more holistic view. When quality improvement is rooted in nursing’s conceptual and theoretical resources, it extends beyond operational improvements. It helps make nursing’s contribution visible, cumulative, and open to further development. The CTE provides nurse leaders with a mechanism for demonstrating nursing’s measurable contributions to patient care, workforce outcomes, and organizational performance.

## 4. A Disciplined Approach to Collaboration

Fawcett was deeply concerned that nursing could lose clarity when entering multidisciplinary work without a strong grasp of its knowledge base. Some readers have found the language of her critique severe. Even so, the leadership implication is constructive and important: collaboration is strongest when each discipline enters with conceptual confidence and professional role clarity. For nurse leaders, partnering with medicine, healthcare administration, public health, and other professions should not require the dilution of nursing’s perspective. On the contrary, multidisciplinary work is enhanced when nursing can articulate what it sees, values, and contributes. Leaders who can do this help create organizations in which nursing is recognized as a full intellectual and interprofessional partner. This matters in international settings, where nurse leaders, especially women, often work within long‐standing provider‐oriented hierarchies. In those settings, discipline‐specific clarity is not an abstract luxury; it is a practical source of confidence, coherence, and agency.

## 5. Theory, Pragmatism, and the Real Work of Leadership

One recurring objection to theory‐guided nursing is that it feels too abstract for the pressures of daily leadership. Fawcett answered that objection by insisting on pragmatic adequacy. A useful conceptual model or theory must be capable of informing practice in ways that are feasible, intelligible, and open to evaluation. That criterion is highly relevant for nursing leadership. Nurse leaders do not need theory for ceremonial purposes. They need it to shape practice models, guide role expectations, inform improvement work and research, support professional development, and clarify what should count as evidence of nursing excellence. Fawcett’s body of work consistently sought to connect conceptual clarity with practical application.

## 6. A Tribute Expressed as Responsibility

The most fitting tribute to Jacqueline Fawcett is not only to praise her scholarship but to respond to her call to action. First, nurse leaders should ensure that professional practice models are explicitly grounded in a nursing conceptual theory. The specific model may vary by context, but the absence of any explicit nursing framework leaves nursing identity vulnerable to diffusion and even perhaps dismissal by other disciplines. Second, quality improvement and evaluation should be designed so that nursing’s contribution can be seen as more than labor. Fawcett’s CTE structure provides a helpful guide. Third, job descriptions, performance appraisal processes, and leadership development pathways should reflect nursing‐specific expectations rather than generic lists. Organizations that value nursing as a profession and discipline make it visible, in part, through the standards by which nurses are appointed, developed, and evaluated. Fourth, nurse leaders should invest in the continuing theoretical education of their workforce and leadership teams. Theory becomes impractical when it is unknown, poorly taught, or disconnected from organizational life. Fawcett spent a career illustrating that this disconnect is not inevitable. Finally, nurse leaders should engage in nursing’s wider intellectual commons, including scholarly dialog, nursing journals, and resources, such as https://nursology.net. The stewardship of nursing knowledge is not the task of universities alone; it is also the work of nurse leaders who shape practice environments and advocate to elevate the nursing discipline.

## 7. Conclusion

Jacqueline Fawcett challenged nursing to be clear about what it knows and courageous about using that knowledge. She also modeled remarkable stewardship of concepts, standards, disciplinary memory, and future possibility. For that, nursing owes her lasting gratitude. For nurse leaders, Fawcett’s legacy is neither ornamental nor remote; it is organizational. The structures leaders design, the outcomes they privilege, the language they authorize, and the standards they enforce all help determine whether nursing appears as a distinct knowledge discipline or only as a highly valued workforce.

As an international network of nursologists, across diverse health systems and cultural contexts, nursing leaders can make nursing’s intellectual contribution more visible. Fawcett gave the discipline conceptual architecture and expected it to be used. To honor her well is to build with it.

## 8. Key Works of Dr. Jacquiline Fawcett

Fawcett, J. (1984). The metaparadigm of nursing: Present status and future refinements. *Image: The Journal of Nursing Scholarship*, 16(3), 84–87. https://doi.org/10.1111/j.1547-5069.1984.tb01393.x.

Fawcett, J. (1992). Conceptual models and nursing practice: *The reciprocal relationship. Journal of Advanced Nursing*, 17(2), 224–228.

Fawcett, J. (2005). Contemporary nursing knowledge: Analysis and evaluation of nursing models and theories (2nd ed.). F.A. Davis.

Fawcett, J. (2017). Applying conceptual models of nursing: Quality improvement, research, and practice. Springer Publishing.

Fawcett, J. (2018‐present). Nursology.net: A website for nurse scholars. https://nursology.net.

Fawcett, J. (2023). Thoughts about the metaparadigm of nursing: Contemporary status and recommendations for evolution. *Nursing Science Quarterly*, 36(3), 303–305. https://doi.org/10.1177/08943184231169770.

Fawcett J. (2024). More Thoughts About the Evolution of the Metaparadigm of Nursing: Addition of Culture as Another Metaparadigm Concept and Definitions of All the Concepts. *Nursing science quarterly*, 37(2), 183–184. https://doi.org/10.1177/08943184231224409.

Fawcett, J., & DeSanto‐Madeya, S. (2013). Contemporary nursing knowledge: Analysis and evaluation of nursing models and theories (3rd ed.). F.A. Davis.

Lee, R. C., & Fawcett, J. (2013). The influence of the metaparadigm of nursing on professional identity development among RN‐BSN students. *Nursing science quarterly*, 26(1), 96–98. https://doi.org/10.1177/0894318412466734.

O’Rourke, M., & Fawcett, J. (2024). More Thoughts About Culture as a Metaparadigm Concept: Rejection of the Reality of Subcultures. *Nursing Science Quarterly*, 37(3), 297–298. https://doi.org/10.1177/08943184241246986.

## Funding

No funding was received for this study.

## Conflicts of Interest

The authors declare no conflicts of interest.

## Data Availability

Data sharing is not applicable to this article as no datasets were generated or analyzed during the current study.

